# Towards a multi-basin SWAT model for the migration of nutrients and pesticides to Puck Bay (Southern Baltic Sea)

**DOI:** 10.7717/peerj.10938

**Published:** 2021-02-25

**Authors:** Paweł Wielgat, Dominika Kalinowska, Adam Szymkiewicz, Piotr Zima, Beata Jaworska-Szulc, Ewa Wojciechowska, Nicole Nawrot, Karolina Matej-Lukowicz, Lidia Anita Dzierzbicka-Glowacka

**Affiliations:** 1 Faculty of Civil and Environmental Engineering, Gdansk University of Technology, Gdańsk, Poland; 2 Institute of Oceanology of the Polish Academy of Sciences, Sopot, Poland

**Keywords:** SWAT model, land use change, nutrient runoff, pesticide runoff, catchment hydrology, Baltic eutrophication, WaterPUCK project, fertilizer, Puck Bay

## Abstract

**Background:**

This paper analyzes the impact of changes in fertilization on crop yields and the runoff of nutrients from a small agricultural catchment (176 km^2^) to a shallow bay, using the SWAT model. Puck Bay is part of the Gulf of Gdansk and belongs to the Baltic Sea. The whole area of Puck Bay (364 km^2^) is protected (Natura 2000) yet despite this it suffers from eutrophication problems due to the relatively minimal depth and difficult water exchange.

**Methods:**

The paper presents a comparison of the calculated yields and the runoff of nutrients and pesticides in the SWAT model, for a small agricultural coastal catchment. Calculations were made for 13 crop scenarios with weather data from 2011 to 2019. For each crop, an agriculture calendar was made. Two variants of fertilization were considered (autofertilization mode and according to the calendar). The nutrient runoff was calculated depending on the adopted scenario. In addition, the fate of selected pesticides was simulated.

**Results:**

Depending on the crop, the annual load of NO_3_into the stream ranged from 0.74 to 3.65 kg ha^−1^. The annual load of organic phosphorous into the stream was between 0.686 and 3.64 kg ha^−1^. This is lower than in the majority of EU or Baltic countries. The surface runoff of dissolved Glyphosate was equal to 286 mg ha^−1^. The annual loads of nutrients from the catchment area are equivalent in both fertilization modes. Regardless of the selected fertilization mode, in addition to the dosage, the form of nutrients is important for the model.

## Introduction

The process of pollution of the Baltic Sea has increased in the 20th century due to urbanization, industrialization and the intensification of agriculture (Boczek, 1978; Rheinheimer, 1998). These processes have been accompanied by changes in drainage basin management, and an increase in the pollution of rivers flowing into the Baltic Sea. Slow water exchange through the Danish Straits (25–30 years) has resulted in the Baltic Sea being one of the world’s most eutrophic seas (European Court of Auditors, 2016; Wojciechowska et al., 2019b). Therefore, its ecosystem is facing many problems including algal and cyanobacterial blooms, andoxygen deficiency zones. The reason for this is the anthropogenic supply of nutrients to the sea via rivers draining adjacent drainage basins. The most important source of nutrient loads is agriculture (45% of the total nitrogen load and 45% of the total phosphorus load) (European Court of Auditors, 2016). The issue of the environmental protection of the Baltic Sea is a subject of international agreements and international law (Declaration, 2013). The quality of and potential threats to the Baltic waters are subjects of many studies pointing to slow improvement (HELCOM, 2015; HELCOM, 2018). Some reduction in emissions can be achieved by rationalizing the use of fertilizers and changing farming methods. However, a large reduction thereof raises concerns about the efficiency and profitability of agricultural production (Pietrzak, 2012; Pastuszak et al., 2015). The so-called Nitrates Directive, introduced into Polish law, imposes limits and deadlines on farmers regarding the use of fertilizers (Dzierzbicka-Głowacka et al., 2019a; Dzierzbicka-Glowacka et al., 2019b). Researchers have analyzed in detail parts of the Baltic ecosystem like bays, lagoons and depths (Polkowska, Astel & Namieśnik, 2005; Glasby & Szefer, 1998; Zima, 2018; Zima, 2019). Under the WaterPUCK project the authors focused on a study of processes occurring in Puck Bay. For this purpose, a model of nutrient transformation in the Bay of Puck was created. The bay model was fed with hydrological data and nutrient load data from the adjacent catchment.

The results presented in the article come from the model which is part of the Integrated information and prediction Web Service WaterPUCK. The aim of the WaterPUCK project is to determine the impact of farms on the water quality of Puck Bay (southern Baltic Sea). The website combines individual mathematical models representing all elements of the water cycle in nature, i.e., the ICM (Interdisciplinary Centre for Mathematical and Computational Modeling–Univesity of Warsaw) meteorological model, the SWAT (Soil and Water Assessment Tool) hydrological model, the MODFLOW groundwater flow model and the EcoPuckBay model based on the POP code. The SWAT model is the link to exchange quantitative and qualitative information between individual elements of the service. Based on spatial data of the catchment area and meteorological data, the SWAT model calculates the surface runoff and flow rate in watercourses, which are the input data for the EcoPuckBay model (point sources of water, nutrients and pesticides). Simultaneously, it generates infiltration information that feeds the MODFLOW model. Before the project, the scientific literature lacked articles about pesticides in the watershed and waters of Puck Bay.

The runoff of nutrients and pesticides from agricultural catchments is studied worldwide using modeling tools (Nixon et al., 1986; Holland, 2004; Beman, Arrigo & Matson, 2005; Bainbridge et al., 2009; Nowik & Dawidowicz, 2011; Thodsen et al., 2017; Casado et al., 2019). In the case of the Baltic Sea, due to the large inflow of river waters and susceptibility to eutrophication, this topic is particularly important (Håkansson, 2004) and has been studied in several adjacent countries (Stålnacke, 1996; Behrendt & Bachor, 1998; Loefgren et al., 1999; Pastuszak et al., 2018). The impact of fertilization restrictions resulting from the Nitrates Directive has been considered with regard to the conditions of the Baltic rivers (Dzierzbicka-Głowacka et al., 2019a; Dzierzbicka-Glowacka et al., 2019b; Wilk, Orlińska-Woźniak & Van, 2019).

The WaterPUCKweb-based service (waterpuck.pl) is used as a decision support tool, enabling local stakeholders (administration and farmers) to predict changes in the quality and quantity of local water resources (Dzierzbicka-Glowacka et al., 2018; Dzierzbicka-Głowacka et al., 2019a; Dzierzbicka-Glowacka et al., 2019b).

The SWAT model is one of the most frequently used tools to study catchment processes.

It allows the anticipation of how changes in the way the basin is managed influence the water balance, the degree of erosion, and pollution with nitrogen and phosphorus compounds, pesticides, bacteria and heavy metals (Wilk, Orlińska-Woźniak & Van, 2019). The model was used to analyze the impact of climate change on crops and on the water balance (Bogdanowicz & Cysewski, 2008; Potrykus et al., 2018; Wojciechowska et al., 2019a), and crop yields were also simulated (Kalinowska et al., 2018). The impact of changes in crop cultivation, the water balance and nutrient runoff were also analyzed (Zawadzki & Bednarek, 1999). SWAT was successfully used to estimate pollutant loads entering the seas (EU Pesticides database—European Commission, 2019; Casado et al., 2019).

Therefore, the SWAT model is also used to optimize agricultural practices (Zawadzki & Bednarek, 1999; Arnold et al., 2013). Depending on the sources of meteorological information, this model can be used for real-time calculations, for forecasting or for the simulation of observed events. Each calculation is based on areas with the same slope, soil type and land use form—HRU (hydrologic response unit). An HRU is a basic computational unit assumed to be homogeneous in its hydrologic response to land cover change (Sawicki & Zima, 1997; Luo & Zhang, 2009; Dzierzbicka-Glowacka et al., 2018).

This paper analyzes the impact of changes in fertilization on crop yields and the runoff of water and nutrients from a small agricultural catchment. The cultivation of crops such as winter wheat, winter triticale, silage corn, winter canola, a mixture of spring cereals, spring barley, potatoes and peas (Pisum) was investigated during a 9-year period from 2011–2019.

Two fertilization modes were compared. First, the autofertilization mode implemented in the SWAT model, which applies nutrients when there is a level of nitrogen stress encountered by the plant. Fertilizer was automatically added by SWAT (according to crop N stress levels) to a maximum yearly load of nitrogen limited by the user settings. The assumed limits of fertilizing were according to the Nitrates Directive. The second mode is called the crop calendar mode, in which mean doses and terms of fertilizing typical for local practices were set. Several cultivation variants were aggregated into scenarios. The purpose of our work was to investigate the following issues:

• Impact of the fertilization mode on the leaching of nutrients and crop yields.

• Impact of crop types and associated typical agriculture practices on the leaching of nutrients and pesticides.

## Materials & Methods

### Study area

**Figure 1:**
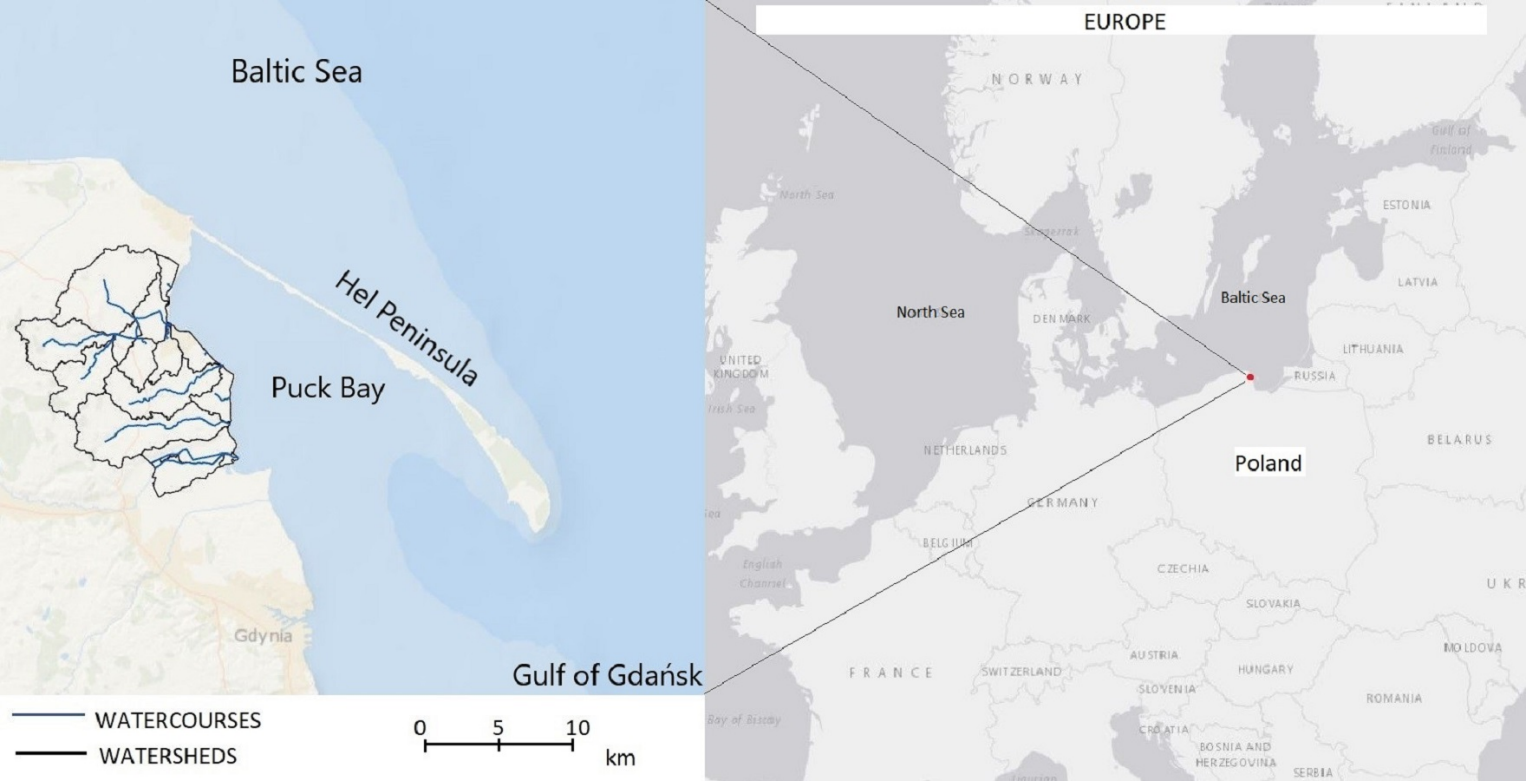
Location of the study area. Map data ©2020 Esri.

The study area is located in northern Poland on the southern coast of the Baltic Sea (Fig. 1). It covers the catchment area of Puck Bay within the Puck commune district with an average population of 107 people per km^2^. The basin area is 176 km^2^. The use of land for agriculture dominates (60%), in addition to forests (29%) and urban areas (11%). This is a young glacial area of varied relief, characterized by a large denivelation (from −0.5 to 113.5 m a.s.l.) and cut by smaller valleys with steep slopes (Potrykus et al., 2018). The bottoms of the valleys contain the beds of rivers, e.g., Gizdepka, Błądzikowski Stream, and Płutnica. The geological structure consists of fluvioglacial sediments, mainly loamy sands and sandy loams interlaced with clay. Peat soils dominate in the valleys. There are two nature reserves in the catchment area: Beka Nature Reserve and Darżlubskie Buki Nature Reserve. Waters from the studied area flow into the Bay of Puck. It is a brackish shallow bay separated from the Baltic Sea by the Hel Peninsula. It is an area of very high biodiversity included in the NATURA 2000 network as a Special Protection Area and Special Area of Conservation (Zima, 2019). Average flows in the analyzed streams are: Gizdepka 0.178 m^3^ s^−1^, Błądzikowski Stream 0.035 m^3^ s^−1^, Płutnica 0.718 m^3^ s^−1^ (Bogdanowicz & Cysewski, 2008). The water quality of each river varies depending on the season of the year, the intensity of vegetation and the agricultural practices (Wojciechowska et al., 2019a). The specific climate of this region, with moderate winters and mild summers, is due to the proximity of the Baltic Sea. In the period 2011–2019 the annual average temperature was about 7.5 °C and the average annual precipitation was 712 mm.

### SWAT model

**Figure 2:**
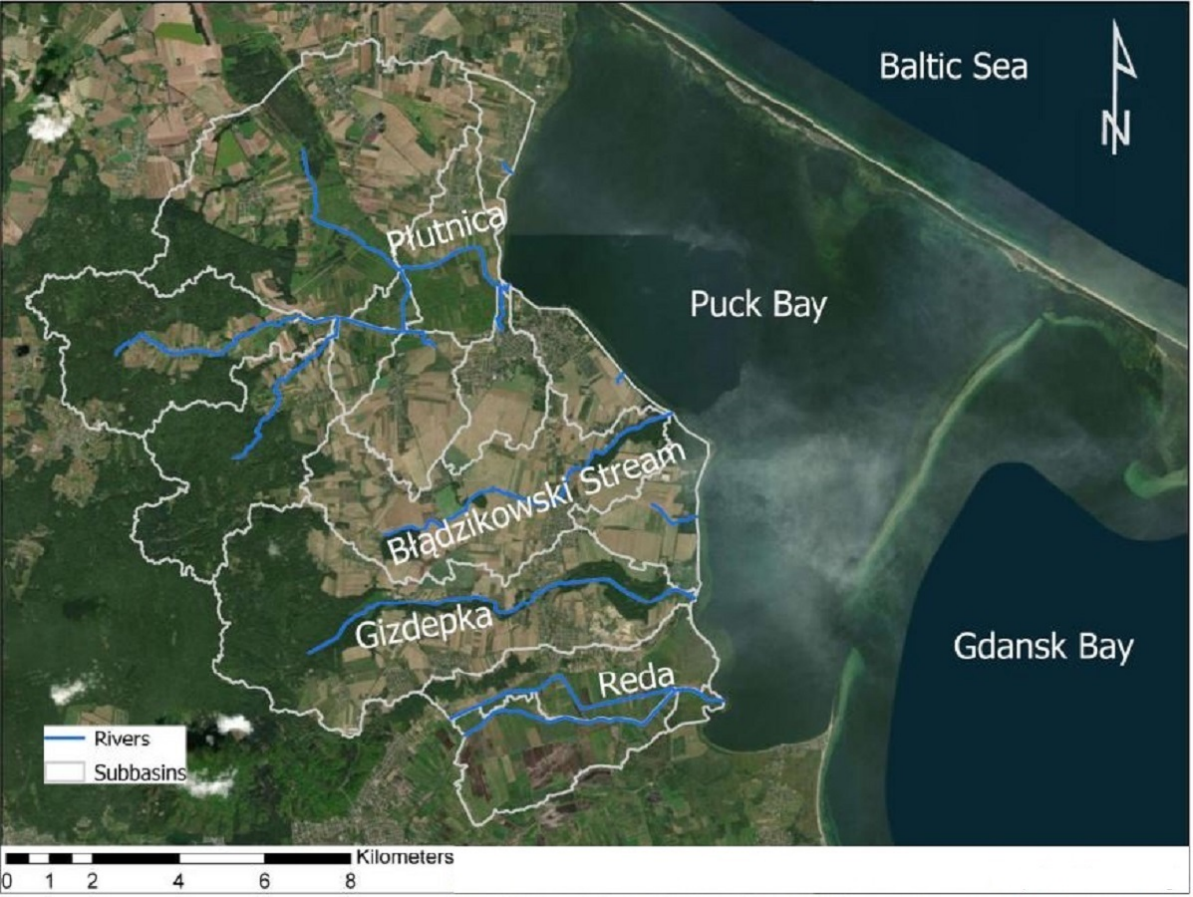
Study area and basins boundaries. Map data ©2020 Google.

The catchment model was created based on a digital terrain model, soil maps and meteorological data. The digital elevation model (DEM) was created from LIDAR data supplied by the Polish Head Office of Geodesy and Cartography. The model was set up using the QGIS SWAT interface. The resolution of the used DEM is 10 m (Kalinowska et al., 2018; Mustafa & Szydłowski, 2020). The basin boundaries determined on the basis of the DEM were compared with topographic and hydrological maps and were also verified by fieldwork (Fig. 2) (Kalinowska et al., 2018; Dzierzbicka-Głowacka et al., 2019a; Dzierzbicka-Glowacka et al., 2019b).

The soil maps were supplied by the Polish Geological Institute. Due to the very fragmented “mosaic” soil, an attempt to accurately reproduce the complicated soil system needed almost 50 different soil profiles. Such a large diversity resulted in the release of over 1000 HRUs, thus the decision was made to generalize. The first step of the generalization was to leave only 2 soil layers in the soil profile. Then 13 types of soil profiles were selected, covering over 95% of the analyzed area. The remaining area was assigned to the main groups based on similarities in the share of the ratio of fraction content (silt, sand and clay fractions) and the saturated hydraulic conductivity. The soil parameters of every soil class were taken to be the average values reported in the Polish literature (Table 1) (Zawadzki & Bednarek, 1999).

**Table 1:** Soil classes used in the model.

Soil type	Area [%]	First soil layer				
		Organic carbon [%]	Clay [%]	Silt [%]	Sand [%]	Rock [%]
Sandy loam	23	4	14	28	58	0
Medium sand/Sandy loam	2	4	4	6	90	0
Medium Sand	1	4	4	6	90	0
Sand	6	5	14	28	58	0
Peat/Medium Sand	7	8	45	45	10	0
Peat	10	37	45	45	10	0
Loamy sand/Sandy loam	30	4	6	11	82	0
Loamy Sand	4	4	6	11	82	0
Clay	18	4	55	5	38	3

The land use map was based on the soil-agricultural map from the Institute of Soil Science and Plant Cultivation, and the digital surface model from the Polish Head Office of Geodesy and Cartography, as well as fieldwork.

**Figure 3:**
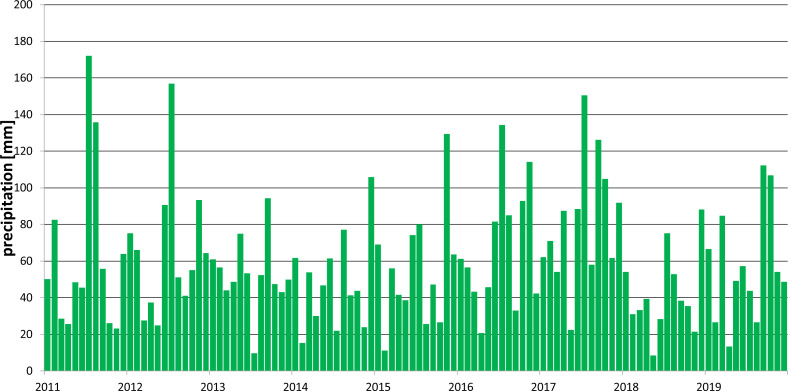
Monthly precipitation in the period 2011–2019.

Meteorological data from the period 2011–2019, used in the simulations, were supplied by the Interdisciplinary Centre for Mathematical and Computational Modelling, University of Warsaw (Fig. 3). In addition, data provided by the Institute of Meteorology and Water Management for the years 2000–2010 were used. This period was treated as the time needed to start the model (warm-up period). Potential evapotranspiration was estimated with the Hargreaves method (Arnold et al., 2013).

The possible full division into HRU units was adopted based on the soil-agricultural map. The slope criterion was not included in our research because crop areas, meadows and pastures located in the flat part of the basin were the most important terrain. The biggest variation in terrain occurs in the western part covered with forests. Finally, a simulation was made consisting of 17 sub-basins and 353 HRUs.

### Crop calendars and fertilizing

Information was obtained from farmers on the type and method of cultivation, dates of agrotechnical operations and doses of fertilizers used in the catchment area (Dzierzbicka-Głowacka et al., 2019a; Dzierzbicka-Glowacka et al., 2019b). Based on the above data, agricultural calendars were created for the most popular crops, containing average agricultural treatment dates and fertilizer doses (Table 2 presents an example crop calendar for winter wheat). The SWAT model allows for defining the doses and dates of fertilization or using the autofertilization mode (Neitsch et al., 2011). The autofertilization mode supplies as many nutrients as the plants need. The auto mode requires only the specification of the type of fertilizer and the start date of autofertilization. In the autofertilization mode, the fertilizers were composed of elemental nitrogen and elemental phosphorus.

**Table 2:** Crop calendar of winter wheat applied in SWAT.

Date	Agricultural practice	Description
27th August	Fertilizer application	Manure fertilizing or urea application on stubble (urea 75 kg ha^−1^)
28th August	Tillage	Disc tilling
08th September	Tillage	Plowing and harrowing
09th September	Fertilizer application	NPK fertilizer (8:24:24 165 kg ha^−1^)
10th September	Planting operation	Sowing
15th September	Pesticide application	Herbicide application (option)
01st March	Fertilizer application	Fertilizing (nitrochalk 218 kg ha^−1^)
05th March	Pesticide application	Herbicide application
20th April	Fertilizer application	Fertilizing (urea 130 kg ha^−1^)
28th April	Pesticide application	Insecticide or fungicide application (option)
15th May	Fertilizer application	Fertilizing (option)
25th May	Pesticide application	Insecticide or fungicide application (option)
01st August	Pesticide application	Desiccant application (option)
15th August	Harvest and kill	Harvest

In order to investigate the effects of different land use on the time variability of loads of nutrients and pesticides in the surface water, 13 scenarios were defined, based on typical agricultural practices for the investigated area. Scenario S1: rotation of main crops in the area (winter canola, winter wheat, silage corn). Scenario S2 is S1 in autofertilization mode. Scenarios S3–S13 assume only one type of crop on agricultural land without any rotation. The crop types are as follows: S3 winter wheat (also representing winter triticale), S4 winter wheat in autofertilization mode, S5 silage corn, S6 silage corn in autofertilization mode, S7 winter canola, S8 winter canola in autofertilization mode, S9 mixture of spring cereals (represented by barley), S10 mixture of spring cereals (represented by barley) in autofertilization mode, S11 potatoes, S12 potatoes in autofertilization mode, S13 peas (Pisum). For silage, dry biomass was considered as the yield. SWAT gives the yields of potatoes as dry mass, thus we assumed a 20% content of dry mass in potatoes in our calculations (Wierzbicka, 2012). Peas occur in just one scenario, without autofertilization, because Polish law limits the fertilization of this plant.

Grassland areas were assigned a permanent plant cover (fescue species, hay cut twice a year). Forests were also modeled as a permanent plant cover. Pine was selected as the representative species.

Autofertilization and doses practiced by farmers did not exceed the standards contained in the Nitrates Directive. Fertilizers available on the local market were added to the implemented fertilizer database, by specifying the necessary parameters in the SWAT model. Farmers mainly used slower acting forms of nitrogen. For natural fertilizers, the SWAT database was used.

### Pesticides

The SWAT model has been used many times to model the surface runoff of pesticides. However, many of the pesticides from the SWAT database have been withdrawn from use in Poland and the European Union, e.g., DDT, atrazine (EU Pesticides database—European Commission, 2019; Barchanska et al., 2017).

The researched area was examined for the presence of pesticides in rivers (Wojciechowska et al., 2019b). Among the detected compounds, pesticides found in the SWAT database (glyphosate amine), as well as new ones not defined in the SWAT database, were determined. Their necessary parameters were adopted based on the literature (Table 3) and then entered into the database used by the model (Pesticide Properties DataBase, 2019; Sawicki & Zima, 1997; Luo & Zhang, 2009; Neitsch et al., 2011; Thomas, Mineau & Juraske, 2011; Arnold et al., 2013; Fohrer et al., 2014).

**Table 3:** Adopted parameters of pesticides used in simulation.

Pesticide	Koc [mg g^−1^]	Wash-off fraction	Half-life foliar [days]	Half-life soil [days]	Water solubility [mg dm^−3^]
Diflufenican (Diflufenican—PPDB: Pesticide Properties DataBase, 2019)	5,504	0.61	32 (Thomas, Mineau & Juraske, 2011)	542	0.05
Metazachlor (Fohrer et al., 2014)	54	0.5	3	9	450
Chlorpyrifos (Luo & Zhang, 2009; Barchanska et al., 2017)	6,070	0.65	3	30	0.4
Glyphosate amine (Neitsch et al., 2011)	24,000	0.6	2.5	47	900,000

### Calibration and validation

Any mathematical model requires calibration in order to reflect reality. In the case of the SWAT model, with a sufficiently large data resource, it is possible to use ready-made programs (e.g., SWAT-CUP) to automate this process. The situation is complicated by the multitude of issues analyzed and the number of parameters included in the calculations, and the related amount of data needed to verify the model. For the needs of the WaterPUCK project, the model was required to deliver data about hydrology, water quality, infiltration, and crop simulation (Dzierzbicka-Glowacka et al., 2018; Dzierzbicka-Głowacka et al., 2019a; Dzierzbicka-Glowacka et al., 2019b). Although monitoring existed at the turn of 2018/2019, in conjunction with several field studies conducted in 2017–2019, the complete data needed for comprehensive model calibration cannot be collected. This is related to the specificity of the area. The collection of data for calibration coincided with a severe drought, during which, in the summer of 2018, almost half of the watercourses dried up. The water levels on the Błądzikowski Stream were so low that most of the time it was impossible to perform hydrometric measurements. Even after heavy rain, the surface runoff was barely noticeable because the non-urbanized drainage basin made up for shortages. Moreover, large slopes, especially in the initial forest fragments of watercourses, affect the rapid response of the drainage basin and the very quick discharge of excess water to Puck Bay.

Due to the lack of sufficient data on stream discharge, it was decided to perform calibration by a manual trial-and-error procedure (Szymkiewicz et al., 2020). For calibration, meteorological data from the years 2000–2009 were used. In the calibration process, the following parameters were adjusted: SCN curve numbers (increased), maximum canopy storage (CANMX, increased in forests), and water in the shallow aquifer returning to the root zone REVAP (increased in forests), as well as the parameters describing pine growth in the “plant.dat” file. The objectives for calibration were the annual groundwater recharge from each HRU (constrained to the range of between 3% and 30% of yearly precipitation), the average annual evapotranspiration and the average annual biomass production in pine forests. A similar approach was successfully used in other studies (Cao et al., 2006; Smarzyńska & Miatkowski, 2016).

## Results

### Calibration and validation

In the present study, the model calibrated for the period 2000–2009 has been used to simulate another time period (2011–2019), which can be considered as a validation of the model. In Table 4 the model outputs for the period 2011–2019 are compared with the reference values obtained from the literature. It can be seen that the agreement is good, except the groundwater recharge, for which the maximum model value significantly exceeds the reference value. This is due to higher precipitation in the years 2011–2019 (from 506 to 979 mm, average 712 mm), compared to the calibration period (average 620 mm). The increased difference between the precipitation and evapotranspiration led to increased surface runoff and groundwater recharge.

**Table 4:** Comparison of model outputs with reference values.

Output Type	Unit	Model value	Reference values
Groundwater recharge	[mm]	57–307	21–216 (Jaworska-Szulc, 2015)
Evapotranspiration of canola	[mm]	381–437	450–495 (Czyzyk & Steinhoff-Wrześniewska, 2017)
Total runoff	[mm]	63–247	47–268 (Bogdanowicz & Cysewski, 2008)
Surface runoff/total runoff ratio	[–]	0.43–0.63 (mean 0.5)	0.5 (Stachý, Czarnecka & Bialuk, 1987)
Pine biomass annual production	[t ha^−1^]	6.8–8.7	6.5–7.5 (Orzełet al., 2005)
Yield: winter wheat	[t ha^−1^]	5.8–7.6	6.4 (Dzierzbicka-Głowacka et al., 2019a; Dzierzbicka-Glowacka et al., 2019b)
Yield: canola	[t ha^−1^]	2.3–3.8	3.8 (Dzierzbicka-Głowacka et al., 2019a; Dzierzbicka-Glowacka et al., 2019b)

The results obtained differ slightly in comparison with the reference data. This is caused by different weather conditions than those reported in the data used for calibration, e.g., higher precipitation.

Information from the monitoring network was used to validate the model. At the mouth of the watercourse an automatic level station was located, then the water level was converted to the flow rate, based on the rating curve. The impact of the cultivation method (type of cultivated plant, type of agrotechnical treatments as well as method of fertilization and plant protection) on the environment, particularly on the quality of the waters of Puck Bay, could be determined after the full agrotechnical cycle, i.e., after a year. Therefore, the results related to the main product based on the SWAT model—the agricultural calculator, are presented in the form of the annual nutrient load.

\begin{eqnarray*}\mathrm{BIAS}=  \frac{\sum _{i=1}^{n}({Q}_{i}^{\mathrm{sim}}-{Q}_{i}^{\mathrm{obs}})}{n} . \end{eqnarray*}

where: *Qi*^sim^—the ith observation; *Qi*^sim^—the ith simulated value; Qmean—the mean of the observed data observation; *n*—the total numer of observations.

Both on a monthly and daily basis, the BIAS parameter = −0.012, which was considered sufficient to compensate for the model error in a longer time unit. This is confirmed by a similar result of the average annual flow: simulated ${Q}_{\mathrm{avg}}^{\mathrm{sim}}=0.171$ m^3^ s^−1^ and observed ${Q}_{\mathrm{avg}}^{\mathrm{obs}}=0.172$ m^3^ s^−1^.

### Water balance

The highest precipitation (979 mm) was recorded in 2017. In this wet year, the surface runoff averaged 238 mm (24% of precipitation), infiltration 293 mm (30% of precipitation), and evapotranspiration 436 mm (45% of precipitation) (Table 5). The proportions of the water balance are less affected by the type of crop and the fertilization method used. For wheat (scenarios S3 and S4), evapotranspiration was the highest and amounted to 47% for calendar fertilization and 48% for autofertilization. For each scenario, the share of the water balance components differs by a maximum of 3% from the average values.

**Table 5:** Water balance in the wet year 2017 and dry year 2018 (precipitations 979 mm and 506 mm), according to the scenarios received from simulations with the SWAT model.

	Surface runoff [mm]		Percolation [mm]		Evapotranspiration [mm]		Surface runoff/total runoff ratio [–]	
Year	2017	2018	2017	2018	2017	2018	2017	2018
Scenario								
S1	230	82	287	102	452	359	0.44	0.56
S2	229	82	286	102	452	359	0.44	0.56
S3	229	65	281	97	458	404	0.44	0.63
S4	226	63	276	97	466	409	0.44	0.65
S5	247	84	303	103	417	353	0.44	0.55
S6	247	84	303	103	417	353	0.44	0.55
S7	239	73	292	98	437	381	0.44	0.58
S8	239	73	292	98	437	381	0.44	0.58
S9	238	71	295	102	435	381	0.44	0.57
S10	238	70	296	102	434	383	0.44	0.56
S11	244	76	299	102	425	368	0.44	0.55
S12	244	76	299	102	425	368	0.44	0.55
S13	242	83	304	103	422	354	0.44	0.54
Average	238	76	293	101	436	373	0.44	0.57

The lowest precipitation was recorded in 2018—506 mm. In this dry year, the surface runoff averaged 76 mm (15% of precipitation), and infiltration 101 mm (20% of precipitation). Plants evapotranspired an average of 373 mm (74% of precipitation) (Table  5). The proportions of the water balance were less affected by the method of fertilization. For wheat (scenarios S3 and S4), evapotranspiration was the highest and amounted to 80% for calendar fertilization and 81% for autofertilization. However, the share in the balance of evapotranspiration in the dry year for a particular crop ranges from 70% (S13 peas) to 81% (S4 winter wheat autofertilization). Depending on the crop, evapotranspiration varies considerably.

The share of individual components of the water balance changes each year depending on the weather conditions. There are slight differences between the water balance in the same year for cultivation in the autofertilization mode, and fertilization specified in the calendar of agricultural practices (for example, wheat in Table 6). For wheat in the autofertilization mode, the share of evapotranspiration is on average 2% (maximum 3%) higher than in the calendar mode. Other components of the balance sheet differ by a maximum of 2%.

**Table 6:** Components of the water balance for winter wheat in two modes of fertilization received from simulations with the SWAT model.

Year	Precipitation [mm]	Surface runoff [mm]	Percolation [mm]	Evapotranspiration [mm]	Surface runoff/ total runoff ratio
S3 winter wheat					
2011	758	165	148	494	0.62
2012	783	149	146	480	0.49
2013	635	113	122	428	0.55
2014	583	77	69	427	0.49
2015	663	101	104	450	0.47
2016	810	172	174	460	0.49
2017	979	229	281	458	0.44
2018	506	65	97	404	0.63
2019	690	116	108	418	0.43
Average	712	132	139	447	0.50
S4 winter wheat autofertilization mode					
2011	758	160	141	505	0.63
2012	783	147	143	486	0.49
2013	635	109	122	439	0.56
2014	583	71	57	444	0.51
2015	663	97	94	458	0.48
2016	810	166	166	472	0.49
2017	979	226	276	466	0.44
2018	506	63	97	409	0.65
2019	690	109	98	432	0.42
Average	712	128	133	457	0.52

### Yields

Average yields are presented in Table 7. The SWAT model calculates the yield for each HRU unit taking into account soil and hydrological conditions. In the scenarios with odd numbers, fertilization was assumed according to the implemented agricultural calendar, while in the scenarios with even numbers, the autofertilization option was used. Biomass was used as the corn yield because only silage corn is grown in the catchment area.

**Table 7:** Comparison of average annual yields (a) dry mass of biomass for silage corn. (b) Dry mass of potato yields.

Average yields comparison [kg ha^−1^]										Yields in investigated area [kg ha^−1^] (Dzierzbicka-Głowacka et al., 2019a; Dzierzbicka-Glowacka et al., 2019b)
Scenario	2011	2012	2013	2014	2015	2016	2017	2018	2019	
S1	6,907	6,428	2,800	7,098	7,084	2,844	6,776	2,869	3,003	–
S2	6,273	6,425	2,792	6,710	7,097	2,839	6,421	3,598	2,922	
S3	6,700	6,534	6,609	6,993	7,022	6,745	6,656	6,180	6,907	6,430
S4	6,635	5,932	5,866	7,587	6,574	7,075	6,475	6,590	7,551	
S5	12,332	8,029	13,255	13,476	8,791	10,958	8,491	15,621	13,034	17,400 (a)
S6	12,547	8,033	13,471	13,533	8,843	10,966	8,656	16,445	13,465	
S7	2,835	2,511	2,804	3,042	2,567	2,844	2,327	3,799	3,041	3,800
S8	2,826	2,507	2,783	3,027	2,563	2,839	2,325	3,766	3,036	
S9	5,609	5,462	5,590	6,167	6,010	5,696	5,012	5,218	6,024	5,240
S10	7,587	6,712	7,545	8,727	8,601	8,044	8,003	8,836	8,078	
S11	4,072	4,046	4,177	4,203	4,101	4,007	4,083	4,250	4,155	5,000 (b)
S12	4,072	4,046	4,177	4,203	4,101	4,007	4,083	4,250	4,155	
S13	2,729	3,109	3,032	2,711	3,598	3,172	2,747	3,539	2,427	3,620

### Nutrient doses

Comparing the total nitrogen doses, the doses given according to the crop calendar are higher for crop rotation S1, silage corn S5, winter canola S7, and potatoes S11 (Appendix A). However, in autofertilization mode, they are higher for winter wheat and spring cereals. The fertilizers used by farmers in the crop calendar are dominated by ammonium. As fertilizers in autofertilization mode, elemental nitrogen and elemental phosphorus were used. The doses of phosphorus applied according to the crop calendar are several times higher than phosphorus fertilization in the autofertilization mode. Organic forms of nitrogen and phosphorus are applied only in crops fertilized with manure by farmers, i.e., S5 silage corn and S11 potatoes.

### Nutrient losses

The largest total nitrogen losses are in silage corn (S5) and potatoes (S11) according to the crop calendar. The highest nitrogen infiltration into groundwater also occurs for these scenarios. It is worth emphasizing that organic nitrogen dominates in the runoff of nutrients. Only during the cultivation of silage corn and potatoes does autumn manure fertilization occur. Losses of nitrate-nitrogen to surface waters are on average three times higher in autofertilized crops that are fertilized only with the nitrate form of nitrogen. The amount of nitrogen uptake by plants is independent of the fertilization mode (Appendix B).

According to the model, the amount of phosphorus uptake by plants is similar for both fertilization modes (Appendix C). The losses of phosphorus and its uptake by plants are similar for different crops, regardless of the fertilization method. According to the model, mainly organic phosphorus is loaded into the stream. The largest phosphorus losses are in silage corn (scenarios S5 and S6).

### Gizdepka River investigation

Despite the fact that with the adopted criteria for the calibration and validation of the model, the reliable time step of the analyzed results is one year, the influence of the type of autofertilization mode on the daily and monthly results was also checked. The paper analyzes the Gizdepka River in detail (Table 8). This is due to the fact that this stream has all the typical features of rivers in the studied area. In addition, it had the best hydrological conditions (no periodic drying).

**Table 8:** Average loads of nutrients in the period 2011–2019.

Scenario	Total N applied [kg ha^−1^]	N-N0_3_ applied [kg ha^−1^]	N-NH_3_ applied [kg ha^−1^]	Organic N applied [kg ha^−1^]	Total P applied [kg ha^−1^]	Mineral P applied [kg ha^−1^]	Organic P applied [kg ha^−1^]	Permissible maximum dose of nitrogen from all sources (“Council Directive 91/676/EEC”) [kg ha^−1^]
S1	110.10	12.74	83.15	14.21	17.16	15.78	1.38	–
S2	105.16	100.56	4.60	0.00	2.11	2.11	0.00	–
S3	100.63	27.04	73.59	0.00	10.94	10.94	0.00	200
S4	139.11	134.52	4.60	0.00	2.11	2.11	0.00	
S5	125.67	8.23	70.06	47.38	27.64	23.05	4.59	240
S6	88.03	83.43	4.60	0.00	2.11	2.11	0.00	
S7	103.61	8.12	95.49	0.00	14.97	14.97	0.00	240
S8	77.56	72.96	4.60	0.00	2.11	2.11	0.00	
S9	56.96	8.12	48.68	0.00	10.98	10.98	0.00	140
S10	122.78	100.61	22.17	0.00	2.11	2.11	0.00	
S11	87.46	8.25	22.35	56.86	24.10	18.60	5.50	180
S12	33.77	29.18	4.60	0.00	2.11	2.11	0.00	
S13	19.14	8.12	11.02	0.00	11.42	11.42	0.00	30

**Figure 4:**
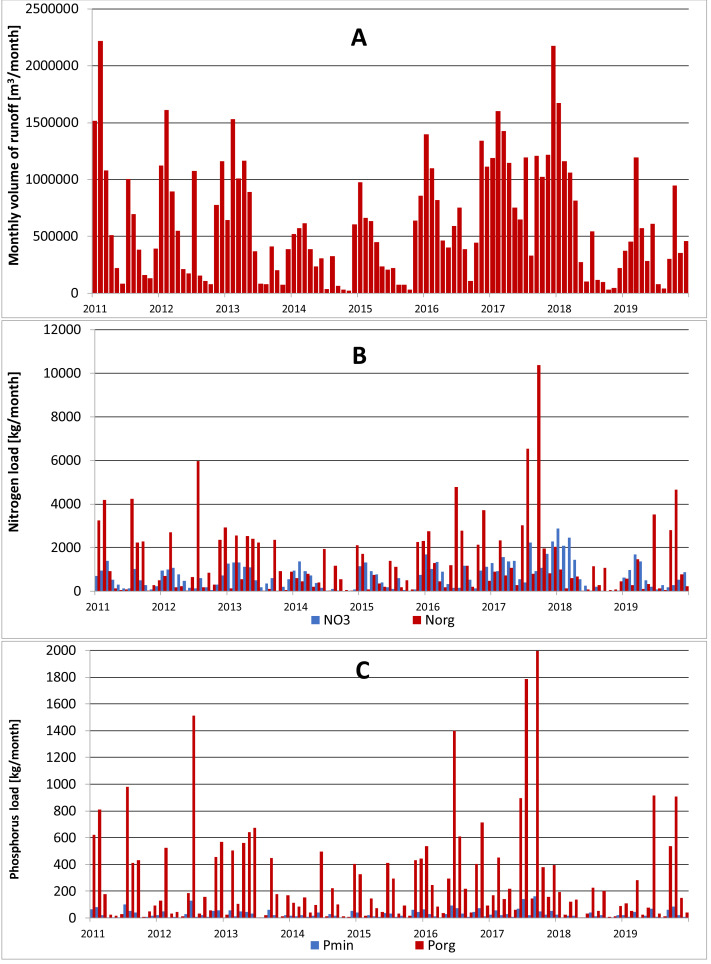
Monthly outflow and nutrient concentration in Gizdepka in the period 2011–2019 for winter wheat (scenario S3). (A) Monthly volume of runoff [m^3^ month^−1^]. (B) Nitrogen load [kg^−1^ month^−1^]. (C) Phosphorus load [kg^−1^ month^−1^].

**Figure 5:**
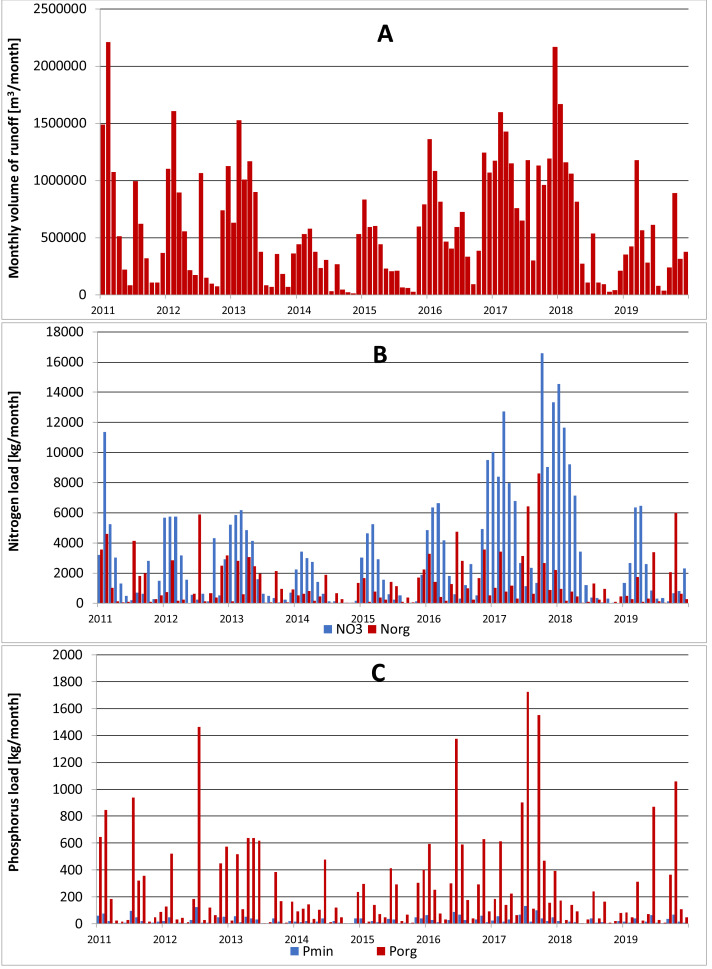
Monthly outflow and nutrient concentration in Gizdepka in the period 2011–2019 for winter wheat in the autofertilization mode (scenario S4). (A) Monthly volume of runoff [m^3^ month^−1^]. (B) Nitrogen load [kg^−1^ month^−1^]. (C) Phosphorus load [kg^−1^ month^−1^].

Analyzing the average monthly nutrient concentrations in the outflow of the Gizdepka, for winter wheat (scenarios S3 and S4) in the autofertilization mode (mainly nitrate-nitrogen), N-NO_3_ concentrations several times higher can be seen. Nitrate values are from 4.3 to 9.1 mg l^−1^ in the autofertilization mode, while in fertilization according to the crop calendar, from 1.3 to 3.2 mg l^−1^. Organic nitrogen concentrations are similar (Figs. 4 and 5). Organic phosphorus losses are comparable between scenarios.

**Figure 6:**
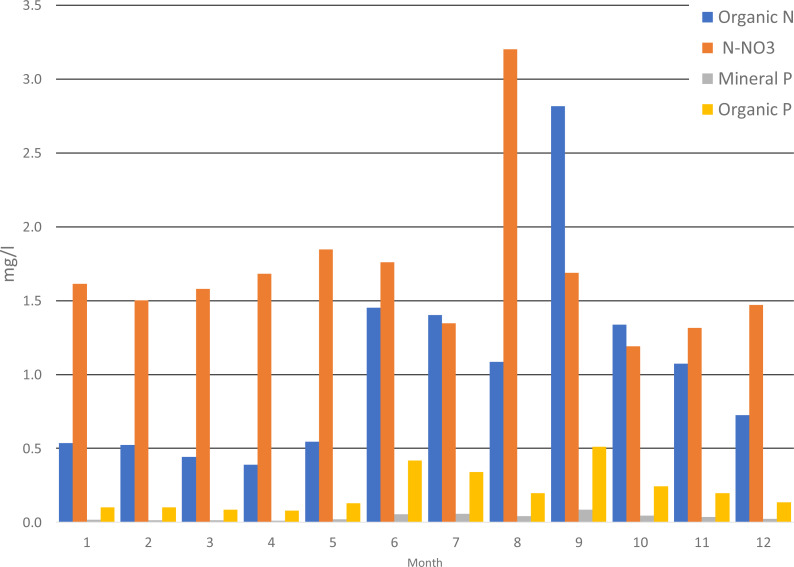
Average monthly concentrations of nutrients in Gizdepka, for 2011–2019, winter wheat in the crop calendar mode (S3).

**Figure 7:**
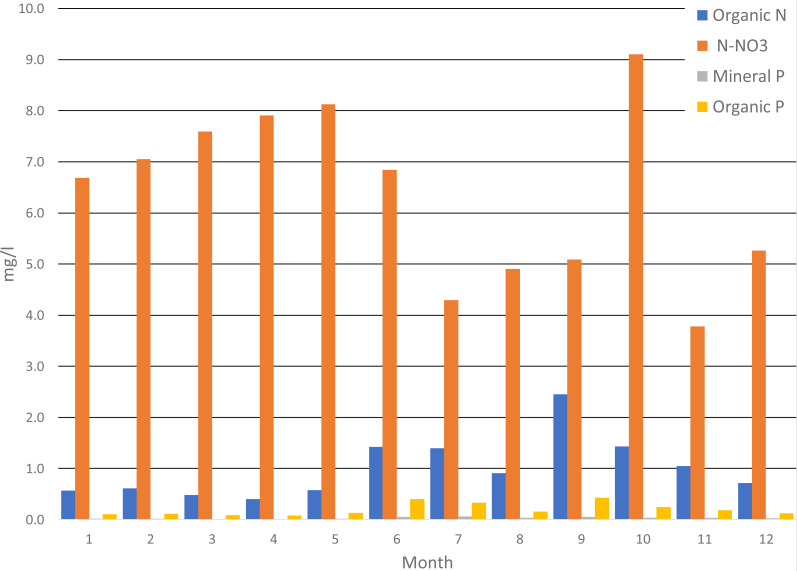
Average monthly concentrations of nutrients in Gizdepka, for 2011–2019, winter wheat in the autofertilization mode (S4).

Similar to the mean values from the 2011–2019 period, the trend of a change in the proportion of the forms of nitrogen observed in the daily outflow is confirmed in 2019, with a significant increase in the concentration of nitrates. A comparison of the monthly sum of nutrient loads clearly indicates the dominance of nitrates in the autofertilization mode with the simultaneous relative compatibility of phosphorus loads (Figs. 6 and 7).

### Pesticides

Not all pesticides used in the analyzed area were modelable. Table 9 presents the results available for the most popular plant protection products: Diflufenican and Glyphosate. Dose refers to the dose of the substance applied to the field, according to the agricultural calendar. The proportion of the dose that was able to react is about 38%. Most of the dose, ranging from 60% to 75%, is destroyed (broken down) before it starts affecting the environment. These losses are caused, among others, by atmospheric conditions (e.g., wind) (Neitsch et al., 2011). There was no significant effect of autofertilization on the reaction of pesticides. In the model, no pesticides were found at the outflow from watercourses, which is consistent with the results of field studies (Pazikowska-Sapota et al., 2020). Only trace amounts of the substance remain on the plant surface (28–38 mg ha^−1^). A large part of the pesticide remains on the ground, amounting to 3% of the substance dose in the case of glyphosate and 85% of diflufenican.

## Discussion

### Water balance

The water balance and the proportions between its components are adequate to the amount of precipitation. In years where rainfall is lower than the average rainfall, the share of evapotranspiration in the balance sheet is much greater. The average rainfall in the studied period was 712 mm. In the analyzed period, an average of 420 mm evapotranspired (60% of the annual average precipitation). An average of 143 mm reached the surface runoff (which is 20% of the average precipitation) and an average of 154 mm infiltrated (21% of the average precipitation—Table 10). By comparison, for another basin in Poland for the years 1963–2010, the average rainfall is 611 mm, while the surface runoff is 106 mm (17% of rainfall) (Banasik & Hejduk, 2012).

### Yields

The average yields for spring crops, represented by barley, are higher in the autofertilization mode scenario (scenario S10) by an average of 2300 kg than when fertilization was based on the agricultural calendar (scenario S9) (Table 7). This difference is most likely due to the type of fertilizer and rapidity of reaction. For the remaining crops, the impact of the fertilization mode is much smaller and without a clear advantage of one fertilization method. The obtained yields are close to the values given in the survey (Dzierzbicka-Głowacka et al., 2019a; Dzierzbicka-Glowacka et al., 2019b).

### Nutrient doses

The greatest difference between fertilization in auto mode and the crop calendar is for spring cereals. In the S9 and S10 scenarios, doses in autofertilization with nitrogen are twice as high. None of the scenarios exceed the maximum doses specified in the Nitrates Directive (Appendix A) (“Council Directive 91/676/EEC of 12 1991 concerning the protection of waters against pollution caused by nitrates from agricultural sources”). In the analyzed area, the use of nitrogenous fertilizers was higher than the average consumption for the whole of Poland and for the Pomeranian Voivodeship. The mean phosphorus fertilizer consumption was higher than in the Pomeranian Voivodeship, but lower compared to the entire country (Dzierzbicka-Głowacka et al., 2019a; Dzierzbicka-Glowacka et al., 2019b).

**Table 9:** Summary of pesticide modeling results.

Scenario/ Pescticide	Dose [mg ha^−1^]	Applied [mg ha^−1^]	Decayed [mg ha^−1^]	Surface runoff disolved [mg ha^−1^]	Surface runoff sorbed [mg ha^−1^]	Final on the plant [mg ha^−1^]	Final on the ground [mg ha^−1^]
S3/Diflufenican	112,000	42,796	73,907	762	542	36	97,176
S4/Diflufenican	112,000	42,796	73,988	716	529	28	97,333
S7/Glyphosate	1,080,000	412,674	814,668	286	3,778	0	35,412
S8/Glyphosate	1,080,000	412,674	814,655	286	3,778	0	35,442

**Table 10:** Average annual water balance for the years 2011–2019 received from simulations with the SWAT model.

Scenario	Surface runoff [mm]	Percolation [mm]	Evapotranspiration [mm]	Surface runoff/ total runoff ratio
S1 rotation crops	144	156	418	0.49
S2 rotation crops autofertilization	144	156	418	0.49
S3 winter wheat	132	139	447	0.50
S4 winter wheat autofertilization	128	133	457	0.52
S5 silage corn	151	161	405	0.50
S6 silage corn autofertilization	151	161	405	0.50
S7 winter canola	144	154	419	0.50
S8 winter canola autofertilization	144	154	419	0.50
S9 mixture of spring cereals	141	152	424	0.50
S10 mixture of spring cereals autofertilization	141	153	423	0.50
S11 potatoes	147	159	412	0.50
S12 potatoes autofertilization	147	159	412	0.50
S13 peas	150	166	401	0.49
Average	143	154	420	0.50

### Nutrient losses

The biggest difference in nitrogen uptake by spring cereals (S9 and S10) may be due to the fact that nitrate-nitrogen from autofertilization is more easily and faster available for spring cereals. Also, the yields of spring cereals (S9 and S10) in the autofertilization scenario are much higher (Table 7). This confirms that nitrate-nitrogen works faster and is better absorbed even during spring water shortages. The diagrams (Figs. 6 and 7) show a higher ratio of nitrates in the surface runoff in the autofertilization scenario, which indicates greater leaching of nitrates from the catchment.

### Gizdepka River investigation

Analyzing nutrient loads, it is plain to see increased values of organic forms (Table 8). This may be related to the fact that the catchment has an agricultural character, arable lands are located on the slopes of moraine hills and in valleys characteristic of a young glacial landscape. These valleys were covered with peat bogs and drained by watercourses. Currently, they are transformed into agricultural areas, becoming a reservoir of soil with a high content of organic carbon (nearly 20% of the catchment area) (Table 1). For the SWAT model, organic nitrogen levels are assigned assuming that the C:N ratio for humic materials is 14:1. Organic phosphorus levels are assigned assuming that the N:P ratio for humic materials is 8:1 (Neitsch et al., 2011). Dependence on the reaction between organic and mineral forms of nutrients as a function of carbon content may be too simplistic (Kemanian et al., 2011). This may explain the high proportion of organic nutrient forms in the runoff.

For the needs of the model, a generalization was made, consisting of the unification of crops in the monoculture in the entire area, classified as farmland. In reality, agricultural land is a mosaic of many crops, including those not included in the analyzed scenarios. A large range of nutrient outflow variability, depending on the adopted scenario, from 872 to 6078 kg N km^−2^ year^−1^ and from 110 to 939 kg P km^−2^ year^−1^ (Table 8), leads to the assumption that the percentage distribution of crops in the catchment area may also have a large effect on the amount of nutrient outflow.

### Pesticides

As part of the fieldwork related to the WaterPUCK project, in 2018 the catchment and adjacent bay were tested for the presence of pesticides. Samples were tested for the presence of 309 substances of different generations, both those currently used and discontinued. Samples of soil, groundwater, water from drainage ditches, watercourses flowing into the bay and water from the bay itself were taken. Only in one sample (Błądzikowski Stream, July 2018), was diflufenican detected with a concentration of 0.13 µg dm^−3^. Substances found in plant protection products were also detected in drainage ditches, in just 17 samples. The majority of them were glyphosate and its derivatives (AMPA), with a maximum concentration of 20 µg dm^−3^ (Gizdepka basin, August 2018), as well as metazachlor with a maximum concentration of 2 µg dm^−3^ (Błądzikowski Stream basin, August 2018). The concentration of other substances was below the limit of quantification (Pazikowska-Sapota et al., 2020).

## Conclusions

In the present study, the impact of changes in fertilization on crop yields and the runoff of nutrients from a small agricultural catchment area (of Puck Bay), based on the SWAT model, were investigated. Several cultivation and fertilization scenarios were considered, leading to the following key findings:

(1) Realistic yield values were obtained for all scenarios, because the agricultural calendars contained the best dates for agricultural treatments, determined on the basis of the guidelines and experience of the surveyed farmers. To obtain accurate results in relation to yields, it is necessary to create an appropriate crop calendar based on local recommendations for agrotechnical operations and their dates. The version of the model based on Heat Units returned unrealistic values that were impossible to calibrate, e.g., wheat harvest in November.

(2) The fertilization mode has no effect on the water balance and does not have a great impact on the crop. The use of the autofertilization mode is more convenient when creating a hydrological model if we do not have to check the outflow of nutrients from the catchment.

(3) If the autofertilization mode is used for nutrient runoff modeling, the type of fertilizer should be carefully selected. The incorrect ratio of nutrient forms contained in the fertilizer, their speed of action and possibility of leaching can affect the results.

(4) The SWAT pesticides database needs updating with new plant protection products.

(5) Manual trial-and-error calibration of relevant model parameters allows satisfactory results to be obtained. It can be successfully used as an interactive overlay in a simple web map application for estimating the water balance, nutrient runoff and yields.

##  Supplemental Information

10.7717/peerj.10938/supp-1 Appendix A Doses of fertilization in the scenarios

10.7717/peerj.10938/supp-2 Appendix B Nitrogen losses for crop and fertilization scenarios

10.7717/peerj.10938/supp-3 Appendix C Phosphorus losses for crop and fertilization scenarios

10.7717/peerj.10938/supp-4 Data S1 Monthly outflow and nutrient concentration in Gizdepka in the period 2011–2019 for winter wheat in the scenario S3 and S4

10.7717/peerj.10938/supp-5 Data S2 Daily outflow and nutients concentration in Gizdepka in 2019

10.7717/peerj.10938/supp-6 Data S3 Water balance for scenarios S1–S13

10.7717/peerj.10938/supp-7 Data S4 Yields for scenarios S1–S13

## Additional Information and Declarations

### Data Availability

The following information was supplied regarding data availability: Raw data are available in the Supplemental Files.
