# Supplementary material for: Towards a multi-basin SWAT model for the migration of nutrients and pesticides to Puck Bay (Southern Baltic Sea)
Source: PeerJ. 2021 Feb 25;9:e10938. doi: 10.7717/peerj.10938 (PMC7916535; doi:10.7717/peerj.10938)
Supplement: Appendix A [file peerj-09-10938-s001.docx]

| Scenario | N applied in fertilizer | Organic N loading to the stream | NO_3_ loading to the stream | NO_3_ loading to the stream from lateral flow | NO_3_ loading to the ground water | NO_3_ percolation past bottom of soil profile | N  uptake  by plant |
| --- | --- | --- | --- | --- | --- | --- | --- |
|  | [kg·ha^-1^] | [kg·ha^-1^] | [kg·ha^-1^] | [kg·ha^-1^] | [kg·ha^-1^] | [kg·ha^-1^] | [kg·ha^-1^] |
| S1 | 110.10 | 22.81 | 1.31 | 0.024 | 10.49 | 30.06 | 169.42 |
| S2 | 105.16 | 22.49 | 3.65 | 0.026 | 9.43 | 28.18 | 167.13 |
| S3 | 100.63 | 5.65 | 1.02 | 0.015 | 3.83 | 13.70 | 184.59 |
| S4 | 139.11 | 5.52 | 3.53 | 0.040 | 12.39 | 43.61 | 191.54 |
| S5 | 125.67 | 53.35 | 1.38 | 0.031 | 13.98 | 38.96 | 139.57 |
| S6 | 88.03 | 51.76 | 3.03 | 0.022 | 7.62 | 23.27 | 147.60 |
| S7 | 103.61 | 29.53 | 1.17 | 0.029 | 15.59 | 42.11 | 164.57 |
| S8 | 77.56 | 29.38 | 2.07 | 0.020 | 6.51 | 20.82 | 158.14 |
| S9 | 56.96 | 24.05 | 0.85 | 0.020 | 7.62 | 22.92 | 98.87 |
| S10 | 122.78 | 21.89 | 3.16 | 0.030 | 11.39 | 34.09 | 175.13 |
| S11 | 87.46 | 49.53 | 0.74 | 0.027 | 13.29 | 36.70 | 87.22 |
| S12 | 33.77 | 47.82 | 1.24 | 0.013 | 5.21 | 16.02 | 82.59 |
| S13 | 19.14 | 31.86 | 1.63 | 0.032 | 14.77 | 39.22 | 174.77 |
