# Supplementary material for: Towards a multi-basin SWAT model for the migration of nutrients and pesticides to Puck Bay (Southern Baltic Sea)
Source: PeerJ. 2021 Feb 25;9:e10938. doi: 10.7717/peerj.10938 (PMC7916535; doi:10.7717/peerj.10938)
Supplement: Appendix B [file peerj-09-10938-s002.docx]

| Scenario | P applied in fertilizer | Organic P loading to the stream | Soluble P loading to the stream | P in percolation | P uptake by plant |
| --- | --- | --- | --- | --- | --- |
|  | [kg·ha^-1^] | [kg·ha^-1^] | [kg·ha^-1^] | [kg·ha^-1^] | [kg·ha^-1^] |
| S1 rotation crops | 17.16 | 2.79 | 0.090 | 0.063 | 25.66 |
| S2 rotation crops autofertilization | 2.11 | 2.77 | 0.073 | 0.063 | 25.42 |
| S3 winter wheat | 10.94 | 0.700 | 0.082 | 0.056 | 24.60 |
| S4 winter wheat autofertilization | 2.11 | 0.686 | 0.070 | 0.054 | 26.08 |
| S5 silage corn | 27.64 | 6.47 | 0.103 | 0.065 | 20.69 |
| S6 silage corn autofertilization | 2.11 | 6.32 | 0.074 | 0.065 | 20.91 |
| S7 winter canola | 14.97 | 3.64 | 0.090 | 0.062 | 31.13 |
| S8 winter canola autofertilization | 2.11 | 3.64 | 0.069 | 0.062 | 31.07 |
| S9 mixture of spring cereals | 10.98 | 2.96 | 0.086 | 0.061 | 18.39 |
| S10 mixture of spring cereals autofertilization | 2.11 | 2.69 | 0.075 | 0.062 | 22.95 |
| S11 potatoes | 24.10 | 6.00 | 0.088 | 0.064 | 15.18 |
| S12 potatoes autofertilization | 2.11 | 5.85 | 0.072 | 0.064 | 15.18 |
| S13 peas | 11.42 | 3.85 | 0.094 | 0.067 | 15.85 |
