# Supplementary material for: Towards a multi-basin SWAT model for the migration of nutrients and pesticides to Puck Bay (Southern Baltic Sea)
Source: PeerJ. 2021 Feb 25;9:e10938. doi: 10.7717/peerj.10938 (PMC7916535; doi:10.7717/peerj.10938)
Supplement: Appendix C [file peerj-09-10938-s003.docx]

| Scenario | Organic N | NO_3_ | Organic P | Mineral P | NH_4_ | NO_2_ |
| --- | --- | --- | --- | --- | --- | --- |
|  | [kg·km^-2^] | [kg·km^-2^] | [kg·km^-2^] | [kg·km^-2^] | [kg·km^-2^] | [kg·km^-2^] |
| S1 | 2212,16 | 939,23 | 407,86 | 26,58 | 56,36 | 0,26 |
| S2 | 2181,97 | 1064,74 | 368,93 | 22,27 | 55,66 | 0,26 |
| S3 | 548,33 | 309,6 | 104,49 | 13,99 | 13,95 | 0,07 |
| S4 | 537,35 | 1372,46 | 97,87 | 12,18 | 13,49 | 0,07 |
| S5 | 5181,65 | 1268,09 | 974,42 | 45,6 | 125,04 | 0,49 |
| S6 | 5027,98 | 814,51 | 846,42 | 35,39 | 121,68 | 0,48 |
| S7 | 2865,39 | 1420,6 | 518,92 | 31,56 | 72,08 | 0,33 |
| S8 | 2850,68 | 633,44 | 472,48 | 25,92 | 71,66 | 0,33 |
| S9 | 2345,06 | 625,92 | 429,23 | 25,9 | 49,62 | 0,14 |
| S10 | 2135,02 | 1215,83 | 365,82 | 22,01 | 45,61 | 0,13 |
| S11 | 4813,91 | 1150,18 | 898,33 | 40,52 | 113,75 | 0,43 |
| S12 | 4647,71 | 432,99 | 797,29 | 33,9 | 110,12 | 0,42 |
| S13 | 3107,04 | 1366,12 | 556,51 | 29,56 | 65,53 | 0,18 |
